# Physiological responses to drought stress of three pine species and comparative transcriptome analysis of *Pinus yunnanensis* var. *pygmaea*

**DOI:** 10.1186/s12864-024-10205-5

**Published:** 2024-03-16

**Authors:** Feng Xiao, Yang Zhao, Xiurong Wang, Xueyan Jian, Yao Yang

**Affiliations:** 1 Institute for Forest Resources and Environment of Guizhou, Key Laboratory of Forest Cultivation in Plateau Mountain of Guizhou Province, College of Forestry, Guizhou University, Guizhou, 550025 China; 2 College of Continuing Education, Yanbian University, Jilin, 133002 China

**Keywords:** *Pinus yunnanensis* var. *pygmaea*, Drought stress, Rehydration, PacBio, Transcriptome, WGCNA

## Abstract

Drought stress can significantly affect plant growth, development, and yield. Fewer comparative studies have been conducted between different species of pines, particularly involving *Pinus yunnanensis* var. *pygmaea* (*P. pygmaea*). In this study, the physiological indices, photosynthetic pigment and related antioxidant enzyme changes in needles from *P. pygmaea*, *P. elliottii* and *P. massoniana* under drought at 0, 7, 14, 21, 28 and 35 d, as well as 7 days after rehydration, were measured. The PacBio single-molecule real-time (SMRT) and Illumina RNA sequencing were used to uncover the gene expression differences in *P. pygmaea* under drought and rehydration conditions. The results showed that the total antioxidant capacity (TAOC) of *P. pygmaea* was significantly higher than *P. massoniana* and *P. elliottii.* TAOC showed a continuous increase trend across all species. Soluble sugar (SS), starch content and non-structural carbohydrate (NSC) of all three pines displayed a "W" pattern, declining initially, increasing, and then decreasing again. *P. pygmaea* exhibits stronger drought tolerance and greater recovery ability under prolonged drought conditions. Through the PacBio SMRT-seq, a total of 50,979 high-quality transcripts were generated, and 6,521 SSR and 5,561 long non-coding RNAs (LncRNAs) were identified. A total of 2310, 1849, 5271, 5947, 7710, and 6854 differentially expressed genes (DEGs) were identified compared to the control (Pp0D) in six pair-wise comparisons of treatment versus control. *bHLH*, *NAC*, *ERF*, *MYB_related*, *C3H* transcription factors (TFs) play an important role in drought tolerance of *P. pygmaea.* KEGG enrichment analysis and Gene set enrichment analysis (GSEA) analysis showed that *P. pygmaea* may respond to drought by enhancing metabolic processes such as ABA signaling pathway, alpha-linolenic acid. Weighted gene co-expression network analysis (WGCNA) revealed *GST*, *CAT*, *LEC14B*, *SEC23* were associated with antioxidant enzyme activity and TAOC. This study provides a basis for further research on drought tolerance differences among coniferous species.

**Supplementary Information:**

The online version contains supplementary material available at 10.1186/s12864-024-10205-5.

## Introduction

*Pinus massoniana* Lamb. (*P. massoniana*), mainly distributed from 21°41′N to 33°56′N and 102°10′E to 123°14′E [1], plays a vital role in soil and water conservation, restoration, and pulpwood production. The broad distribution and diverse uses make *P. massoniana* pivotal for sustainable forestry production and ecological environment construction [2]. Compared with *Pinus elliottii* (*P. elliottii*), *P. massoniana* grows slower in the early stage (10–15 years) and faster in the later stage (> 15 years) [3]. *P. elliottii* is an important timber and resin-producing species grown worldwide [4]. In China, due to its strong adaptability, high resin yield, low resin crystallization rate, and high turpentine content, *P. elliottii* has become one of the main tree species for resin-tapping [5]. *P. yunnanensis* suffers from genetic degradation, with high proportion of twisted trunk, leading to the emergence of mutated variants such as the *Pinus yunnanensis* var. *pygmaea* (*P. pygmaea*) [6]. *P. pygmaea* maintains its dwarfism even growing in a suitable habitat [7]. Compared to angiosperms, gymnosperms are characterized by higher vulnerability to subsequent droughts and more evident lagged drought effects (“legacy effects”) [8]. The above-mentioned three pine species have a widespread distribution and significant economic value in China. However, there is limited research on the response to adversities and stress among different pine tree species.

Frequent natural abiotic stresses such as low temperature [9], drought [10], and low phosphorus [11] make us realize that the strength of tolerance is a key factor that affects and limits the normal growth into forests, and directly determines the survival rate and stand retention rate after afforestation. Due to climate change, drought will become increasingly frequent, severe, and longer [12, 13]. Drought significantly reduces the photosynthetic rate, the accumulation of biomass, and inhibits plant growth [14, 15]. Plants respond to drought stress through morphological and structural changes, expression of drought-resistant genes, hormone synthesis, and osmotic regulation substances [16]. Under continuous drought conditions, trees close stomata or reduce leaf area to reduce water lose [17]. For instance, *P. palustris* responded to drought treatment with greater stomatal control of plant water loss [18]. Plant hormones, especially ABA, are considered to be closely associated with drought responses. Stomatal closure is controlled by both active (abscisic acid(ABA)-mediated closure) and passive (hydraulic closure) regulation [19]. ABA-dependent stomatal closure is among the most rapid plant reactions to water stress [20]. ABA accumulates during drought stress and rapidly decreases upon rehydration [8]. Long-term water shortage induces the formation of stress memory that maintained a higher level of ABA accumulation in trees grown in more arid long-term conditions [21]. Osmoprotectants such as total soluble sugars and proteins were early accumulated in primed plants during the stress [22]. Meanwhile, antioxidant protection systems perform an essential function during drought stress [23]. Many cell components are damaged by reactive oxygen species (ROS) that are generated as a result of decreases in photosynthesis [24]. Antioxidant enzymes, such as catalase (CAT), peroxidase (POD) and superoxide dismutase (SOD) play a central role in plant adaptation to environmental changes [25]. Osmolarity-related compounds and antioxidant enzyme activity will increase under drought stress [26]. The high level of antioxidative enzyme activity is important for plants to tolerate stress for keeping good growth state and forming good quality [27]. Plants can adapt to prolonged drought by remodeling transcriptomes [28]. The recovery ability after rehydration is important for the successful adaptation of plants to arid environments [29]. Recovery of *P. massoniana* seedlings is more likely to be inhibited when plants experience increasing drought stress [30].

Third-generation Pacific BioSciences (PacBio) single-molecule real-time (SMRT) sequencing does not need to interrupt RNA fragments, and direct reverse transcription can be used to obtain full-length cDNA [31, 32]. Full-length transcripts that do not require assembly can be used to detect genes with AS events, APA events, lncRNAs and fusion transcripts [33]. Pacbio SMRT sequence is widely used to obtain long reads and assemble a high quality reference transcriptome. PacBio-SMRT sequencing can provide a high-quality reference transcriptome for non-genomic species. RNA-seq is one of the most important and practical methods for exploring conifers genes [34, 35]. RNA-seq helps to provide differential gene expression information under different stress conditions, time periods, and genotypes [36]. Resilience strategies differed among tree provenances: wet forests showed higher growth resistance to drought, while dry forests presented faster growth recovery [37]. The response to stress pressure varies inconsistently among different species. *Pterocarya stenoptera* and *P. elliottii* seedlings developed different adaptive strategies in response to continuous flooding [38]. Among *P. elliottii*, *P. palustris* and *P. taeda*, *P. elliottii* is the least drought-tolerant [39]. There has been extensive research on the drought response of *P. massoniana*. *P. pygmaea* has narrower niche and more adaption for extreme ecological conditions [40]. However, fewer comparative studies have been conducted between different species of pines, particularly involving *P. pygmaea*. The physiological and molecular mechanisms of *P. pygmaea* are unclear.

In this study, the needles of three pines (*P. pygmaea*, *P. elliottii*, *P. massoniana*) were used as the research material to measure the antioxidant enzyme activity and physiological responses after drought and rehydration. By combining PacBio SMRT seq and RNA-seq, the molecular responses of *P. pygmaea* were studied to identify key genes and pathways. This study provides a foundation for breeding drought-resistant pine trees, and comprehending the physiological and molecular mechanisms underlying drought response.

## Results

### Physiological responses of three pine species under drought and rehydration

**Fig. 1:**
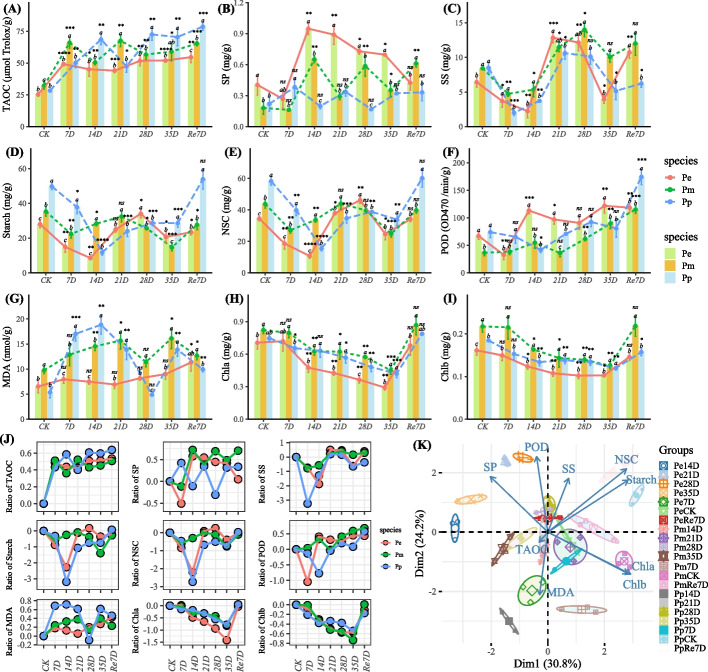
The physiological changes of three pines under drought and rehydration. **A** Total antioxidant capacity (TAOC); **B** Soluble proteins (SP); **C** Soluble sugars (SS); **D** Starch; **E** Non-structural carbohydrate (NSC); **F** Peroxidase (POD) activity; **G** Malondialdehyde (MDA) content; **H** Chlorophyll a (Chla); **I** Chlorophyll b (Chlb); **J** The relative change rate of indicators compared to CK at different time points (ratio = (Value_(point)_—Value_(CK)_)/Value_(CK)_); **K** PCA analysis. Note: In (A–I), 7-35D represented drought treatment after 7 D, 14D, 21D, 28D, and 35D. Re7D represented 7 D after rehydration; error bars indicated the standard deviation, the number of seedlings measured for each species was *n* = 3; the left Y-axis represented value of physiological traitors, “CK” group of each species was set as a reference to compare the mean by the Student's t-Test, “ns” means no difference,”*” *P* ≤ 0.05;”**” *P* ≤ 0.01;”***” *P* ≤ 0.001;”****” *P* ≤ 0.0001. A least significant difference test (LSD) was used at a probability level of 0.05 to verify the significance between species at sample point

Physiological indicators were measured in the three pine species (*P. pygmaea*, *P. elliottii*, *P. massoniana*) under prolonged drought stress. As the drought time lengthened, total antioxidant capacity (TAOC) showed a continuous increase across all species. At the Re7D, *P. pygmaea* had significantly higher TAOC than *P. massoniana* and *P. elliottii* (Fig. 1A). The soluble protein (SP) content of *P. elliottii* exhibited an initial decrease followed by an increase, with levels significantly higher than those of *P. massoniana* and *P. pygmaea* at several points between 14 and 35 days of drought stress (Fig. 1B). The soluble sugar (SS) content (Fig. 1C), starch content (Fig. 1D), and Non-structural carbohydrate (NSC) (Fig. 1E) of all three pines displayed a "W" pattern. Initially, they decreased under prolonged drought stress, then increased, and finally decreased again before recovering after rehydration. After rehydration, *P. pygmaea*'s POD enzyme activity was higher than the other two species (Fig. 1F), and malondialdehyde (MDA) content was lower than *P. elliottii* and *P. massoniana* at Re7D (Fig. 1G). During drought, the content of Chlorophyll a (Chla) and Chlorophyll b (Chlb) decreased continuously (Fig, 1H&I). After the drought treatments on three pine species, compared to the control (0d), TAOC showed an increasing trend, while chla and chlb exhibited a decreasing trend (Fig. 1J). Principal component analysis (PCA) (Fig. 1K) indicated that Re7D samples of the three pines were relatively similar, while the control (CK) samples of the pines were closed to each other.

### PacBio SMRT sequencing data analysis

**Fig. 2:**
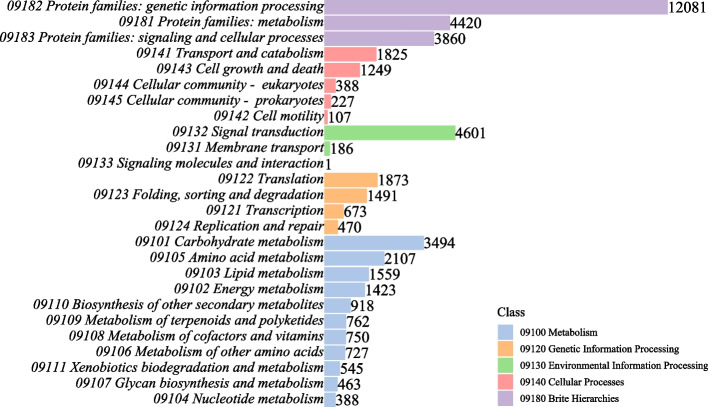
KEGG functional classifications of high-quality transcripts

The RNA extraction quality and concentration of all samples was satisfactory (A260/280 = 2.0–2.2; A260/230 = 1.8–2.2; 28S/18S = 1.4–2.7; Rin ≥ 8.0). The mixed samples of the *P. pygmaea* were sequenced using the PacBio SMRT platform. For PacBio SMRT-seq, a total of 33,095,230 subreads (75.1 Gb of bam file) with 1200.8 mean read length were obtained, 360,635 circular consensus (CCS) reads, 274,760 full-length Non-concatemer (FLNC), and 139,075 consensus reads were acquired. A total of 50,979 transcripts encoded by 42,919 genes were obtained after removing redundancy and clustering by CD-HIT. The mean length of the transcripts was 1,468.5 bp. 6,521 SSR candidates and 5,561 long non-coding RNA (LncRNA) candidates were identified. The Benchmarking Universal Single-Copy Orthologs (BUSCO) completeness score for nonredundant transcripts showed the final transcriptome was 59.3% (951) complete and 2.6% (41) fragmented. A total of 41,198 (80.81%) transcripts were annotated using the NR database and exhibited homology with known proteins of various species, including *Picea sitchensis* (36.09%), *Asparagus officinalis* (8.69%), *Amborella trichopoda* (5.05%). Using Uniprot, 41,295 (81%) transcripts were annotated. Identification of transcription factors found that the number of *bHLH* family was the most (2650), followed by *NAC *(1832) (Table S2). Furthermore, 16,574 transcripts were identified in the Kyoto encyclopedia of genes and genomes (KEGG) database and grouped into 265 KEGG pathways, which were divided into five broad categories: genetic information processing (12,081), signal transduction (4601), and metabolism (4420) (Fig. 2).

### Screening and functional annotation of differentially expressed genes (DEGs)

**Fig. 3:**
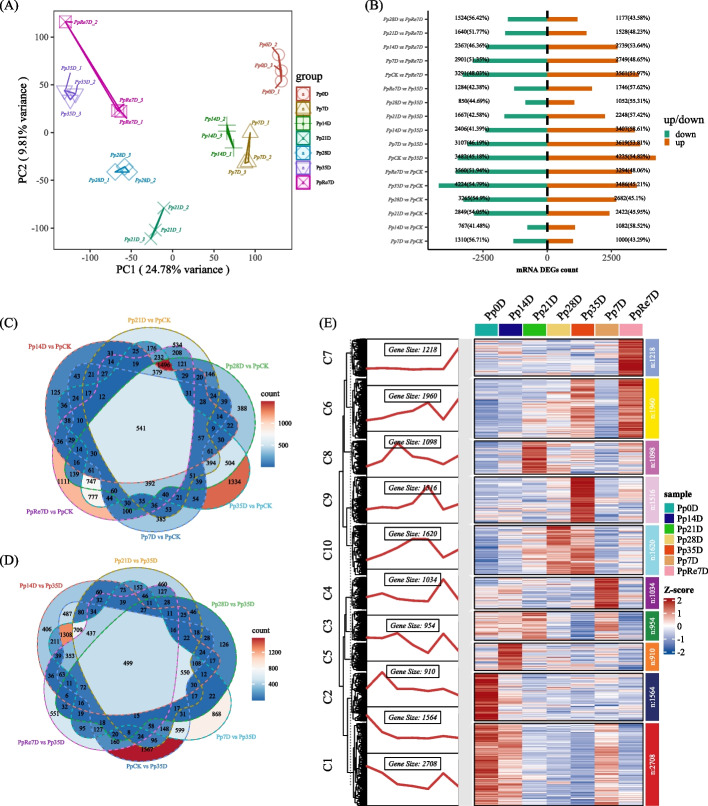
Transcriptome data analysis and differential expression gene quantity analysis. **A** PCA; **B** Summary of the identified DEGs; **C** Venn diagram of DEGs between PpCK and other groups; **D** Venn diagram of DEGs between Pp35D and other groups; **E** The clustered and heatmap analysis of DEGs

For Illumina RNA-seq, a total of 45 Gb raw reads were obtained, the Q20 and Q30 scores for all samples of clean data aboved 99%, and the average content of GC was 44.54%, indicating good sequencing quality. Then, the clean reads were successfully mapped back to the full-length transcripts of PacBio SMRT. PCA analysis showed that samples within the same group were close to each other, indicating good repeatability (Fig. 3A). Based on the DEGs screening criteria, DEGs among all pairwise combinations were identified (Fig. 3B). Among them, compared with the PpCK (Pp0D), the numbers of DEGs between Pp14D and Pp35D were 1,849 (1082 (58.52%) up-regulated and 767 (41.48%) down-regulated) and 7,710 (3,486 (45.21%) up-regulated genes and 4,224 (54.79%)). There were 541 DEGs commonly shared between all groups compared with the PpCK (Fig. 3C), and 499 DEGs commonly shared between all groups compared with the Pp35D (Fig. 3D). Cluster analysis of the DEGs shared by PpCK revealed that genes within C1 (2,708 gene) and C2 (1,564 gene) were highly expressed in PpCK, but lowly expressed in all other samples which were related to drought stress. Genes within C6 (1,960 gene) and C7 (1,218 gene) were highly expressed in PpRe7D and maybe related to rehydration (Fig. 3E).

### Short time-series expression miner analysis (STEM) and gene set enrichment analysis (GSEA)

**Fig. 4:**
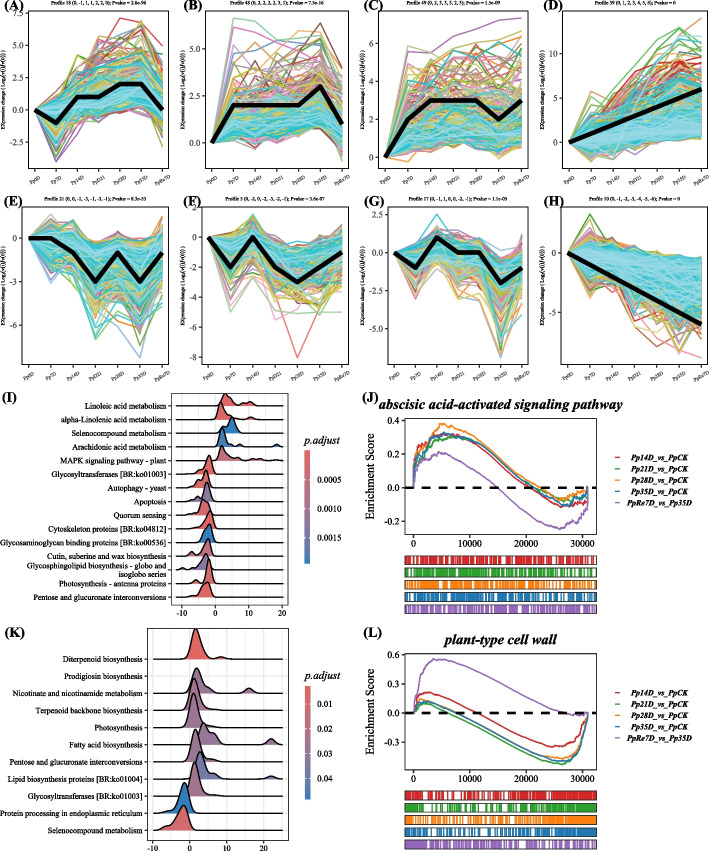
STEM analysis of the gene expression pattern and GSEA analysis. Note: In **A-H**, each subfigure was a significant profile. The upper part of the subfigure showed the number and corresponding trend of the modules. Black line represented the profile trendline. Each line represented the relative expression trend of a gene. In **I**, GSEA of KEGG pathway between Pp35D and Pp0D; In **J**, GSEA of the “abscisic acid-activated signaling pathway” among the four groups; In **K**, GSEA of KEGG pathway between PpRe7D and Pp35D; In **L**, GSEA of the “plant-type cell wall” among the four groups

STEM analysis identified 8 significant profiles. The Profile 18 (0, -1, 1, 1, 2, 2, 0) showed a high expression level in the later stages of drought (Fig. 4A). The Profile 48 (0, 2, 2, 2, 2, 3, 1) showed a highest expression level at Pp35D (Fig. 4B). The Profile 49 showed a lowest expression level at Pp14D and Pp35D (Fig. 4C). The Profile 39 (0, 1, 2, 3, 4, 5, 6) showed a up-regulated trend (Fig. 4D), the KEGG enrichment showed that “Glycolysis / Gluconeogenesis” (ko00010), “Galactose metabolism” (ko00052), “Alpha-Linolenic acid metabolism” (ko00592), “Pyruvate metabolism” (ko00620), Metabolism of xenobiotics by cytochrome P450 (ko00980), “Flavonoid biosynthesis” (ko00941), and others pathways were enriched. The GO enrichment showed “fatty acid catabolic process” (GO:0006633), “fatty acid beta-oxidation” (GO:0006635), “positive regulation of hormone metabolic process” (GO:0032352), “hormone metabolic process” (GO:0042445), and “response to oxygen radical” (GO:0000305) were enriched.

The Profile 21 (0, 0, -1, -3, -1, -3, -1) showed a lowest expression at PpD21 and PpD35 (Fig. 4E). The Profile 5 (0, -2, 0, -2, -3, -2, -1) showed a lowest expression at PpD28 (Fig. 4F). The Profile 17 (0, -1, 1, 0, 0, -2, -1) showed a lowest expression at PpD28 (Fig. 4G). The Profile 10 (0, -1, -2, -3, -4, -5, -6) showed a downregulated trend (Fig. 4H). The KEGG enrichment of the Profile 10 genes showed that “Cutin, suberine and wax biosynthesis” (ko00073), “Photosynthesis proteins (ko00194), Photosynthesis” (ko00195), “Photosynthesis-antenna proteins” (ko00196), “Porphyrin and chlorophyll metabolism” (ko00860), “beta-Alanine metabolism” (ko00410) and others pathways were enriched.

The enrichment of GO terms and KEGG pathways by gene set enrichment analysis (GSEA) in the transcriptome, comparing Pp35D with Pp0D and PpRe7D. Results of the GSEA between Pp35D and Pp0D showed that “Linoleic acid metabolism”, “Alpha-Linolenic acid metabolism”, “Selenocompound metabolism”, “Arachidonic acid metabolism” and “MAPK signaling” pathway were upregulated in the Pp35D, “Cytoskeleton proteins”, “Glycosaminoglycan binding proteins”, “Photosynthesis-antenna proteins” were downgulated in the Pp35D (Fig. 4I). The GO term of “abscisic acid-activated signaling pathway” was enriched in two drought term (Pp28D, Pp35D) compared with PpCK (Fig. 4J). Compared with the Pp35D, “Diterpenoid biosynthesis”, “Prodigiosin biosynthesis, “Nicotinate and nicotinamide metabolism”, “Terpenoid backbone biosynthesis”, “Photosynthesis, “Fatty acid biosynthesis”, “Fatty acid biosynthesis”, “Lipid biosynthesis proteins”, “Glycosyltransferases” were upregulated in the PpRe7D (Fig. 4K). The GO term of “plant-type cell wall” was inhibited under drought conditions and activated after rehydration (Fig. 4L).

### Identification of co-expression module

**Fig. 5:**
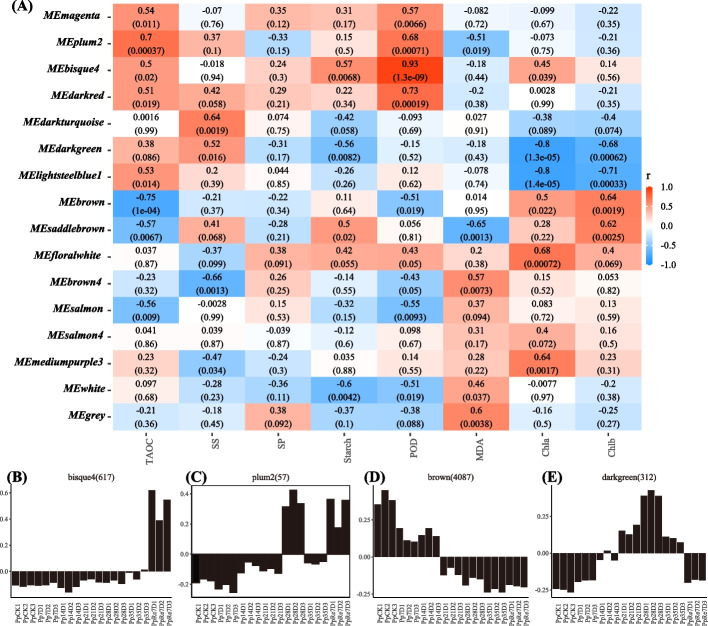
Weighted gene co-expression network analysis of the genes **A** Correlated heatmap of the adjacency of modules; **B-E** The eigengene expression of bisque4, plum2, brown, darkgreen module, respectively. Note: In a, each row represented a module, the color and number of each cell represented the correlation coefficient between modules and traits, the top number in the cell represented the correlation coefficient, the bottom number represented the *p*-*value*, the module name was shown on the y-axis, the color scale on the right shows module-trait correlation from −1 (blue) to 1 (red)

All filtered DEGs were retained for WGCNA analysis. The analysis identified 15 co-expression modules (expect for grey module) (Fig. 5A), of which bisque4 module was positive correlation with POD (correlation coefficient (*r*) = 0.93, *p* = 1.3 × 10^–9^), while salmon and white module was negative correlation with POD, with *r* of -0.55 (*p* = 0.0093), -0.51 (*p* = 0.019), respectively. The brown module was negative correlation with TAOC (*r* = -0.75, *p* = 1 × 10^–4^). The plum2 module was positive correlation with TAOC (*r* = 0.7, *p* = 0.00037). The genes within the bisque4 module showed relatively lower expression during the term of rehydration (Fig. 5B). The genes within the plum2 module showed a higher expression at Pp28D and PpRe7D (Fig. 5C). The genes within the brown module showed a lower expression after Pp14D (Fig. 5D), while the darkgreen module genes showed a highest expression at Pp28D (Fig. 5E).

**Fig. 6:**
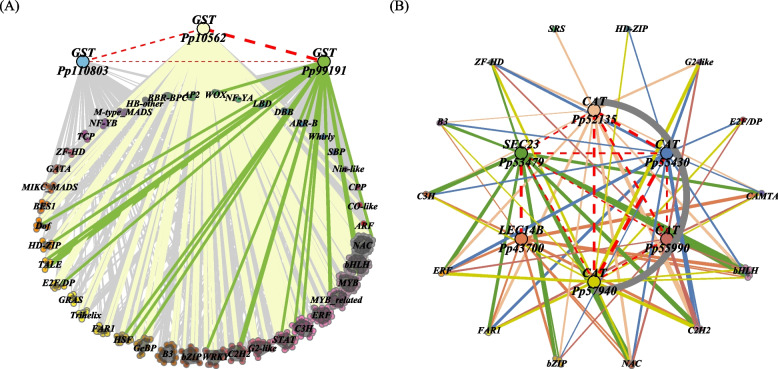
Partial of the co-expression network of module bwtween structure genes and transcription factors. **A** bisque4 module; **B** plum2 module. Note: In **A**, the top three genes which filled with different colors represented structural genes, with 'Pp' abbreviating 'D1_transcript_'. The TF families were shown below. In **B**, 'Pp' abbreviated 'D1_transcript_', and the genes which filled with different colors in the middle represented structural genes. The arc encompasses genes of the same class, with TF connected on the periphery. In both **A** and **B**, the points within each gene family were randomly distributed inside the circle, with the radius of the circle proportional to the number of distribution points. The outermost points were calculated and connected using the chull function of grDevices R Package. The connections between structural genes were mapped in 'red' color with a dashed linetype. The thickness of the lines were mapped based on the weight value

In the bisque4 module (Fig. 6A), three *GST* gene (D1_transcript_10562, D1_transcript_110803, D1_transcript_99191) were enriched. Four *Catalase *(CAT) genes (D1_transcript_52135, D1_transcript_55430, D1_transcript_55990, D1_transcript_57940), *protein transport SEC23 *(D1_transcript_53479), *LEC14B homolog *(D1_transcript_43700) were enriched in the plum2 module (Fig. 6B).

### Validation of RNA-seq expression analysis

**Fig. 7:**
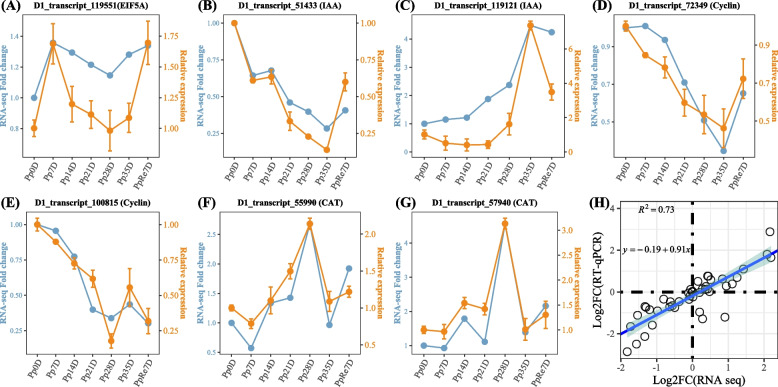
qRT-PCR verification of DEGs. Seven genes were randomly chosen for qRT-PCR validation. Data was shown as mean ± SD (standard deviation). The bars represented the standard deviation (*n* = 3). The *actin* was used as an internal control to normalize the expression data. Note: In **A-G **the left of the double axis represented RNA-seq, the right of the double axis represented the qRT-PCR; In **H**, correlation between the relative expression of transcriptome and qRT-PCR

To validate the accuracy of RNA-seq data, seven genes were randomly selected for the quantitative real-time PCR (qRT-PCR) analysis. The qRT-PCR results showed that the expression patterns of genes were similar to their results in the RNA-seq (Fig. 7A-G). A strong positive correlation (*r* = 0.91, *R*^*2*^ = 0.73) was obtained by a linear regression analysis (Fig. 7H), suggesting that the transcriptome data was reliable.

## Discussion

In the present study, we investigated the response of *P. pygmaea*, *P. elliottii*, and *P. massoniana* seedlings to drought and subsequent recovery. We measured changes in the antioxidant system and oxidative stress levels, and evaluated the physiological plasticity of these species following rehydration.

Plant varieties with drought resistance have armed with higher antioxidant enzyme activities [41]. Drought induced an increase in TAOC which is proportional to the duration and intensity of water deprivation [27]. The results showed that the TAOC of *P. pygmaea* was significantly higher than that of the other two pines (Fig. 1A), which may have stronger drought tolerance. After rehydration, the TAOC of the three species remained at a relatively high level. This maintenance of high TAOC after rehydration is similar to other plants [42]. The SP of *P. elliottii* exhibited an initial decrease followed by an increase, with levels significantly higher than those of *P. massoniana* and *P. pygmaea* at several points between 14 and 35 days of drought stress (Fig. 1B). Above-ground tissues of drought trees showed increases in both sucrose and starch [43]. NSC are major substrates for plant metabolism and have been implicated in mediating drought-induced tree mortality [44]. NSC are important “buffers” to maintain plant functions under drought conditions, the seedlings under the severe and moderate drought conditions tended to invest newly fixed C to starch storage and hydraulic repair instead of growth [45]. Once drought ceased, C allocation to storage was still prioritized at the expense of growth [46]. Severe drought treatment largely reduced photosynthetic carbon assimilation [47]. Plants may have allocated more NSC from source organs to roots [48]. After rehydration, the content of starch and NSC in *P. pygmaea* was higher than that of the other species, indicating a strong recovery ability (Fig. 1B). The occurrence of drought stress can affect the decrease of photosynthetic parameters and chlorophyll fluorescence [27]. The SS content, starch content, and NSC (Fig. 1C-E) of all three pines displayed a "W" pattern. Drought stress lasted for 15 days caused obvious photosynthesis inhibition in *Atractylodes lancea* [27]. In general, drought significantly reduce Chla and Chlb content [49]. During drought, the content of Chla and Chlb decreases continuously (Fig. 1H&I). The activities of CAT and POD increased under drought stress [50], the activities of POD and SOD increased significantly compared with the control [51]. After rehydration, *P. pygmaea*'s POD activity was higher than the other two species (Fig. 1F), and MDA content was lower than *P. elliottii* and *P. massoniana* at Re7D (Fig. 1G). Most antioxidant enzyme indicators still fail to reach the control level after 7 days of rehydration, which may indicate that the 7 day period is insufficient to offset the impact of drought. Other study have shown that, the recovery of *P. massoniana* MAD needs to continue to decrease to the control level after 15 days of rehydration [52]. Long term drought still requires a period of recovery, this recovery time may be determined by the characteristics of species.

Through the PacBio SMRT-seq, approximately 75G of raw bam file was obtained, a total of 50,979 high-quality transcripts were generated. The number of DEGs gradually increased with the deepening of drought and decreased after rehydration, which was consistent with previous research [53]. Continuous drought can affect the reduction of photosynthetic related genes in plants [54], *Trachycarpus fortunei* reduced its light-capturing ability and composition of its photosynthetic apparatus, thereby reducing photosynthesis [55]. Drought induced a common reduction in the level of DEGs and differentially expressed proteins associated with photosynthesis [56]. The DEGs in Profile 10 (Fig. 4H) involved in "Cutin, suberine and wax biosynthesis” (ko00073), “Photosynthesis proteins (ko00194), Photosynthesis” (ko00195), “Photosynthesis (antenna proteins)” (ko00196), “Porphyrin and chlorophyll metabolism” (ko00860), “beta-Alanine metabolism” (ko00410). "Starch and sucrose metabolism", “porphyrin and chlorophyll metabolism”, “photosynthesis (antenna protein)” and other pathways were enriched in the drought stress response of *Nitraria tangutorum* [57]. DEGs were enriched in soluble sugar-related and cell wall-related processes in *P. massoniana* under drought [58]*.* Drought stress inhibited the synthesis of Chla, Chlb and photosynthesis in *Phoebe bournei* [59]. In our study, the genes on the “Porphyrin and Chlorophyll metabolism” (ko00860) pathway were suppressed. “Glycolysis/Gluconeogenesis” (ko00010), “Galactose metabolism” (ko00052), “alpha-Linolenic acid metabolism” (ko00592), “Pyruvate metabolism” (ko00620), “Metabolism of xenobiotics by cytochrome P450” (ko00980), “Flavonoid biosynthesis” (ko00941) and others pathways were enriched in Profile 39 (0, 1, 2, 3, 4, 5, 6) (Fig. 4D). TFs are widely recognized to be involved in plant growth, development, and biotic/abiotic stress responses. Four TFs (*MYB*, *AP2/ERF*, *WRKY*, and *bHLH*) mainly appeared in the roots and stems at D15 and D25 of drought stress in *P. elliottii* [60]. Through the STEM analysis, TFs with continuously increasing gene expression trends in Profile39 (Fig. 4D) were identified, resulting in a total of 1064 TFs. Among them, *bHLH* (116), *NAC* (89), *ERF* (76), *MYB_related* (76), *C3H *(62) genes accounted for the majority. This indicates that *bHLH*, *NAC*, *ERF*, *MYB_related*, and *C3H* TFs play crucial roles in drought tolerance of *P. pygmaea*.

GSEA showed that “Linoleic acid metabolism”, “Alpha-Linolenic acid metabolism” were upregulated in the Pp35D (compared to the Pp0D). “Cytoskeleton proteins”, “Glycosaminoglycan binding proteins”, “Photosynthesis-antenna proteins” were downgulated in the Pp35D (Fig. 4I). Alpha-Linolenic acid significantly increases under drought stress [61]. Compared with fully watered *P. taeda* seedlings, levels of some unsaturated fatty acids significantly reduced in needles of under water stress, such as palmitelaidic acid, erucic acid, and alpha-linolenic acid [62]. The needles of *P. massoniana* seedlings may respond to drought mainly through regulating ABA and JA hormone-related pathways [63]. ABA can accumulate 10- to 30-fold in plants under drought stress relative to unstressed conditions [64]. ABA signaling pathway is an important signal, induces stomatal closure and inhibition of transpiration under drought stress [65, 66]. ABA-responsive genes were obviously upregulated at drought treatment for 7 days [67]. The GO term of “abscisic acid-activated signaling pathway” was enriched in two drought term (Pp28D, Pp35D) compared with PpCK (Fig. 4J). Two *SNRK2* (sucrose non-fermenting 1-related protein kinase 2) genes, five *PYL* genes, eight *PP2C* genes, seven *ACAA1* genes and three *ABF* genes were enriched in the core enrichment of the between Pp35D and PpCK. PYR/PYLs are ABA receptors functioning at the apex of a negative regulatory pathway that controls ABA signaling by inhibiting PP2Cs [68]. *PYLs/PP2Cs* heterodimer blocks substrates binding to *PP2Cs*, and thus releases *SnRK2s* whose kinase activity is formerly inhibited by *PP2Cs* through physical interaction and dephosphorylation, finally allows the plants to adapt to the stress conditions [69]. *TaABFs*-regulated *PYL* gene (*TaPYL1-1B*) exhibited higher ABA sensitivity, photosynthetic capacity and water-use efficiency, all contributed to higher drought tolerance than that of wild-type [70]. Alpha-linolenic acid mostly participated alleviated drought stress through JA signaling [71]. As a precursor to the synthesis of JA, α-linolenic acid in lipid metabolism is related to drought stress [71]. Different strains of tobacco seedlings were enriched in functions about alpha-linolenic acid, and arginine and proline metabolisms under drought [72]. In the alpha-linolenic acid pathway, two *alcohol dehydrogenase* (*ADH1*) genes, *five Lipoxygenase* (*LOX*) genes were enriched. *LOX* play essential roles in responding to biotic and abiotic stresses through oxidizing various lipids [73]. Overexpression of oriental melon *CmLOX13* improved drought tolerance in *Arabidopsis* [74]. Compared with the Pp35D, “Diterpenoid biosynthesis”, “Prodigiosin biosynthesis”, “Nicotinate and nicotinamide metabolism”, “Terpenoid backbone biosynthesis”, “Photosynthesis”, “Fatty acid biosynthesis”, “Fatty acid biosynthesis”, “Lipid biosynthesis proteins”, “Glycosyltransferases” were upregulated in the PpRe7D (Fig. 4K). These findings suggest that *P. pygmaea* may respond to drought by enhancing metabolic processes such as ABA signaling pathway, alpha-linolenic acid, which enhances overall antioxidant capacity.

The results of WGCNA showed that bisque4 module was positively associated with POD (Fig. 5A). In the bisque4 module, “Flavonoid biosynthesis” (ko00941) and other pathways were enriched. Three *GST* gene were enriched in the bisque4 module. Overexpression of *MsGSTU8* of *Medicago sativa* in *Nicotiana tabacum* resulted in maintained chlorophyll content, increased antioxidant enzyme activity, and soluble sugar levels after salt-alkali treatments [75]. These findings suggest that *GST* genes play an important role in scavenging reactive oxygen species and enhancing antioxidant enzyme activity. The brown module was negative correlation with TAOC. The genes in the brown module were significantly inhibited in expression after drought. “Cytoskeleton proteins” (ko04812), “Photosynthesis-antenna proteins” (ko00196), “Phenylpropanoid biosynthesis” (ko00940), “Porphyrin and chlorophyll metabolism” (ko00860) and others were enriched. This indicates that drought can inhibit the synthesis of genes involved in photosynthesis, chlorophyll, and phenylpropanoid biosynthesis. The plum2 was positively associated with TAOC. Four *CAT* genes, protein transport *SEC23, LEC14B homolog* were enriched in the plum2 module. The cultivars Coral Orange, Tendersweet and Solar Yellow were tolerant to drought stress, which was supported by their higher transcript levels of *CAT*, *SOD* genes [76]. The drought tolerant cultivars have higher accumulated of *CAT* gene [77]. *LEC14B* (JK340642) had significantly higher level of expression in drought-tolerant genotype (Tifway) than in drought-sensitive genotype (C299) at 10 d of drought, but no significant differences were detected between two genotypes at 5 of drought [78]. These suggests that *CAT, LEC14B* genes may enhance antioxidant capacity and suppress ROS. *Sec23*/*Sec24* are important cytosolic components of vesicle coat protein II (COPII) [79]. *SEC23* is responsible for transporting newly synthesized proteins and lipids from the endoplasmic reticulum (ER) to the Golgi apparatus in cells, *Steptoe SEC23* (A0A287NVF2) was down regulated under N deficiency, drought stress or both stress [80]. In our study, *two SEC23* (D1_transcript_5780, D1_transcript_28060), two *SEC13*, two *SEC24*, three *SEC31* were enriched in the brown modules, showing a relatively low expression trend in the later stages of drought (Fig. 5D). But not all *SEC* genes expression were inhibited, the *SEC23* (D1_transcript_53479) was one of the plum2 module and one of the Profile 39 (0, 1, 2, 3, 4, 5, 6) genes, showed a upregulated trends under drought stress, meaning *SEC23* in *P. pygmaea* play important roles in regulating both trafficking and plasma membrane stability.

Considering the significance of the selected module hub genes, we will proceed to validate the functions of the related genes through gene cloning and overexpression. Furthermore, it is worth noting whether the drought-tolerant type of *P. pygmaea*, when used as a rootstock for grafting with *P. massoniana* scions, leading to changes in phenotypes such as scion height and resistance in the grafted plants. This study will provide a valuable direction for seedling nursery establishment, rootstock breeding, and reduction of artificial production costs in conifer orchards.

## Conclusions

Different species exhibit inconsistent drought tolerance. Physiological assessments of drought and rehydration responses in three pine species (*P. pygmaea*, *P. elliottii* and *P. massoniana*) indicate that *P. pygmaea* exhibited a stronger drought response and a strong recovery ability under prolonged drought conditions. High-quality transcriptome of *P. pygmaea* was obtained using PacBio SMRT sequencing. Comparative transcriptome analysis indicates that drought suppresses pathways related to photosynthesis and chlorophyll synthesis metabolism. *P. pygmaea* may respond to drought by enhancing metabolic processes such as ABA signaling pathway, alpha-linolenic acid, which enhances overall antioxidant capacity. WGCNA revealed *GST*, *CAT*, *LEC14B*, *SEC23* were associated with antioxidant enzyme activity and TAOC. qRT-PCR analysis correlated well with the data obtained by RNAseq. This study provides valuable insights into the drought response and regulatory processes of *P. pygmaea*.

## Materials and methods

### Plant material and drought treatment

*P. pygmaea* exhibit a clear absence of apical dominance with an inconspicuous main trunk. The base is multi-branched, clumped, with a height range of 40–50 cm to 1–2 m. Leaves grow short and upright while its seed cones cluster together [3]. Tracheid length of *P. pygmaea* is shortened, resin canals increase and more or less concentrate in the late wood [7]. P. elliottii trees grow to 30 m tall. Through early seed resource investigation and collection, the seeds of half-sib progeny of *P. pygmaea*, *P. massoniana* and *P. elliottii* were obtained from the high-quality half-sibling progeny of Ma'anshan Seed Garden (26°16’ N and 107°31’ E), Duyun City, Guizhou Province, China. The seeds were planted in the greenhouse, using a humus: yellow loam soil mixture (1:3) as the soil medium. In order to obtain a detailed genetic background of *P. pygmaea*, different parts of a 5-year *P. pygmaea *were collected for PacBio SMRT sequencing, including the roots, needles, ovulate strobilus, and staminate strobilus. We selected three types of annual pine trees as the experimental subjects. Prior to the drought treatment, the three types of pine trees were irrigated normally. Sampling was conducted between 8:00–9:00 in the morning, mature needles were collected from the stem tips every seven days from new plants, labeled as 0D, 7D, 14D, 21D, and 28D during the drought treatment. A total of 35 days between the cessation of irrigation and the end of the severe drought. When the drought has lasted for 35 days, some seedlings began to die, and the drought treatment is stopped (labeled as 35D). After rehydration, the seedlings were left to grow for seven days. At the end of this period, samples were collected and labeled as Re7d. Three biological replicates were used for each treatment.

### Determination of physiological indexes

The TAOC of needles was determined using 2,2'-azino-bis[3-ethylbenzothiazoline-6-sulfonic acid] diammonium salt (ABTS^•+^) method [81]. The frozen samples (about 2.0 g) were homogenized with 15 mL of 50% methanol and centrifuged at 10,000 rpm for 20 min at 4 °C, the supernatant was collected for ABTS analysis. SP content was measured by Coomassie Brilliant Blue G-250 staining [82]. Fresh needles (0.1 g) were cut and fully ground in phosphate buffer. Then added 5 mL of Coomassie Brilliant Blue G-250 solution. The mixture was then fully mixed and incubated for 2 min at room temperature. The absorbance at 595 nm was measured. SS content was determined using phenol method [83]. Fresh needles (0.5 g) was extracted in 10 mL of ddH_2_O for 20 min at 90 °C and centrifuged at 4000 rpm for 5 min. A combination of 1 mL of sample extract, 0.5 mL of anthrone-ethylacetate reagent, and 5 mL of concentrated sulfuric acid was homogenized and boiled for 5 min, and then rapidly cooled. The absorbance of the mixtures was detected at 630 nm. Starch content was measured by the anthrone colorimetric [84]. NSC was defined as the sum of soluble sugar and starch concentrations [85]. POD enzyme activity was determined using guaiacol method. The reaction mixture was as follows, 50 mmol/L Tris–HCl buffer (pH 7.0) containing 0.1 mmol/L EDTA, 10 mmol/L guaiacol, 5 mmol/L H_2_O_2_ and 100 μL enzyme extract. After the enzyme solution was added to the reaction system, it was immediately incubated at 34 °C in a water bath for 3 min. The changes in absorbance values were measured at 470 nm. One unit of POD activity was expressed as units (μmol guaiacol decomposed per minute) per mg of fresh weight (FW). MDA content was determined using thiobarbituric acid (TBA) method [86]. Each fresh sample (about 0.5 g) was homogenized with 4 ml of 0.1% 0.1% (w/v) trichloroacetic acid (TCA) in ice bath and centrifuged at 4000 rpm for 10 min at 4 °C, for every 1 ml aliquot, 1 ml of 20% (w/v) TCA comprised of 0.5% (w/v) TBA was added. The mixture was heated at 95 °C for 30 min and then cooled immediately before centrifugation again. The absorbance of the supernatants was detected at 532, 450, and 600 nm. Fresh needles were cut into pieces and merged in 80% (v/v) acetone in the dark at room for 24 h to extract chlorophyll. Chla, Chlb were calculated by ultraviolet–visible spectrophotometer at 663, 646 and 470 nm [87]. Three biological replicates were used for the above experiments.

### PacBio library construction,sequencing, and annotation

Total RNA was extracted according to the instruction manual for Trizol reagent (Invitrogen, Carlsbad, CA, USA). RNA integrity was assessed by agarose gel electrophoresis, RNA purity (OD260/280 and OD260/230), concentration and RNA integrity number (RIN), 28S/18S were detected using a NanoDrop 2000 spectrophotometer and an Agilent 2100 (Agilent Technologies, Santa Clara, California, USA). The mRNA was enriched with Oligo (dT) magnetic beads. After all RNA samples were qualified, the total RNA from different tissues was mixed at equal ratios. After the library was qualified, it was sequenced base on the PacBio platform (Menlo Park, CA, USA). The raw bam file had been deposited in the NCBI SRA database (BioProject *acc. *PRJNA994856). The raw seqencing subreads were filtered using SMRTLink v8.0 (https://www.pacb.com/support/software-downloads/) with default parameters. The CCS was obtained by merging the subreads from the same polymerase reads, CCS were obtained by self-correction. FLNC sequences refer to a kind of CCS that contains both 5’ and 3’ primers and poly-A tail, without chimeric reads. After clustering the FLNC reads using the IsoSeq module, the consistent sequences in each cluster were further corrected. Finally, high-quality full-length transcripts (HQ) with an accuracy greater than 99% and low-quality (LQ) transcripts were obtained. Using CD-HIT software [88] to remove redundant sequences in transcripts and cluster the transcript sequences. BUSCO v5.3.2 [89] was used to assess the assembly quality, the BUSCO dataset of 1603 predefined single-copy genes dedicated to gymnosperms [90] were download and used. The transcripts were annotated using the NCBI non-redundant protein (NR) database, the Swiss-Prot protein (http://www.expasy.org/sprot/), the Eukaryotic Orthologous Groups (KOG) database (http://www.ncbi.nlm.nih.gov/COG/), the Pfam database (http://pfam.sanger.ac.uk/), Kyoto Encyclopedia of Genes and Genomes (KEGG) (http://www.genome.jp/kegg, cut-off *e-value* ≤ 1e-5) pathways, and the eggNOG database (http://eggnog5.embl.de/#/app/home) [91]. The SSRs were identified using the MISA software [92]. To identify long non-coding RNA (LncRNA), the transcripts with coding potential were filtered using four computational approaches, including the Coding Potential Assessment Tool (CPAT), Coding-Non-Coding Index (CNCI), Coding Potential Calculator (CPC), and Pfam protein structure domain analysis (Pfam).

### RNA-seq library preparation, sequencing

The needles samples of different stages of *P. pygmaea*'s response to drought and rehydration were collected. The total RNA was isolated using the Trizol Reagent (Invitrogen, Carlsbad, CA, USA). RNA purity, concentration and integrity were assessed using agarose gel electrophoresis, NanoDrop 1000 spectrophotometer (NanoDrop Technologies Inc., Wilmington, DE, USA) and Agilent 2100 (Agilent Technologies, Santa Clara, California, USA). The mRNA was enriched with Oligo (dT) magnetic beads. The mRNA was added to fragmentation buffer and cut into short fragments. Using mRNA as a template, cDNA was reverse-transcribed using six-base random primers. The double-stranded cDNA samples were purified, end-repaired, added with poly(A) tails, and then ligated to sequencing adapters to create cDNA libraries, qualified libraries were sequenced using the NovaSeq 6000 system with paired-end reads. The raw reads generated from Illumina sequencing were deposited in the NCBI SRA database (BioProject *acc. *PRJNA972125).

### Expression quantification, identification of DEGs

Fastp v0.20.1 was used for for quality control [93]. The non-redundant transcripts which obtained from Pacbio SMRT sequencing was used as background transcripts. Bowtie2 v2.4.3 [94] was used to align the sequenced paired-end transcriptomic data. The gene expression level was determined according to the fragments per kilobase of transcript per million mapped reads (FPKM) by the RSEM v1.2.12 [95]. The DEGs were identified based on the counts using DESeq2 v1.40.1 R package [96]. DEGs screening threshold was set to adjusted *p-value* cutoff < 0.05 and |foldchange|> 2. Gene Ontology (GO) and KEGG enrichment analysis were performed by the clusterProfiler v4.8.1 R package [97]. STEM software [98] was used to perform the trend clustering analysis. GSEA based on GO terms and KEGG pathways were conducted by the clusterProfiler, gene sets were considered statistically significant at an *p.adjust* of 0.05. Gene co-expression networks were constructed using the WGCNA v1.72.1 R package [99]. All the DEGs were filtered with at least one sample FPKM value greater than 5.

### Quantitative real-time PCR analysis

In order to verify the accuracy of transcriptome, seven genes were randomly selected for qRT-PCR. The Primer Premier 5.0 software was used to design the primers (Table S1). The first strand of the cDNA was synthesized from total RNA. qRT-PCR was performed on a real-time CFX96 Touch Real-Time PCR System (Bio-Rad, USA). Each 50 μL reaction included: 25 μL of 2X SYBR® Green PCR Master Mix, 1μL of Forward /Reverse primer (10 μM), 2.0 μL of cDNA template, and 21 μL of RNase-free water. The PCR reaction conditions were as follows: preheating at 95 °C for 30 s, 40 cycles of heat denaturation at 95 °C for 5 s, annealing at 60 °C for 34 s. The *actin* gene was used as a reference gene. The gene relative expression levels were calculated according to the 2^−ΔΔCt^ method [100].

### Statistical analysis

The variance of the data was analyzed using the R v4.2.3 software, and the significance threshold was set at *p* < 0.05. LSD was used at a probability level of 0.05 to verify the significance between species. PCA was performed using the FactoMineR v2.8 R package [101].

### Supplementary Information

**Supplementary Material 1. **

## Data Availability

The raw reads generated from Illumina sequencing have been deposited in the NCBI SRA database (BioProject *acc.* PRJNA972125). The raw bam file from PacBio SMRT sequencing has been deposited in the NCBI SRA database (BioProject* acc*. PRJNA994856).

## Abbreviations

*P. massoniana*: *Pinus massoniana*
*P. pygmaea*: *Pinus yunnanensis* var. *pygmaea*
ABA: Abscisic acid
*CAT gene*: Catalase
CCS: Circular consensus sequencing
r: Correlation coefficient
Chla: Chlorophyll a
Chlb: Chlorophyll b
CPAT: Coding Potential Assessment Tool
CNCI: Coding-Non-Coding Index
CPC: Coding Potential Calculator
DEGs: Differentially expressed genes
FPKM: Fragments per kilobase of transcript per million mapped reads
FLNC: Full-length non-concatemer
GSEA: Gene set enrichment analysis
HQ: High-quality full-length transcripts
KEGG: Kyoto encyclopedia of genes and genomes
LncRNA: Long non-coding RNAs
LOX: Lipoxygenase
LSD: Least significant difference test
MDA: Malondialdehyde
PacBio: Pacific BioSciences
PCA: Principal component analysis
POD: Peroxidase
qRT-PCR: Quantitative real-time PCR
RIN: RNA integrity number
ROS: Reactive oxygen species
SD: Standard deviation
SP: Soluble protein
SS: Soluble sugar
SMRT: Single-molecule real-time
STEM: Short time-series expression miner
TAOC: The total antioxidant capacity
WGCNA: Weighted gene co-expression network analysis

## References

[1] Fan F, Wang Q, Li H, Ding G, Wen X (2021). Transcriptome-wide identification and expression profiles of masson pine WRKY transcription factors in response to low phosphorus stress. Plant Mol Biol Rep.

[2] Quan W, Ding G (2017). Root tip structure and volatile organic compound responses to drought stress in Masson pine (Pinus massoniana Lamb.). Acta Physiol Plant..

[3] Xiao F, Zhao Y, Wang X, Yang Y (2022). Targeted metabolic and transcriptomic analysis of pinus yunnanensis var. pygmaea with loss of apical dominance. Curr Issues Mol Biol..

[4] Du B, Luan Q, Ni Z, Sun H, Jiang J (2022). Radial growth and non-structural carbohydrate partitioning response to resin tapping of slash pine (Pinus elliottii Engelm. var. elliottii). J Forestry Res..

[5] Lai M, Zhang L, Lei L, Liu S, Jia T, Yi M (2020). Inheritance of resin yield and main resin components in Pinus elliottii Engelm at three locations in southern China. Industrial crops and products..

[6] Cai N, Xu Y, Li G, Deng L, Li W, Wang D (2016). Research status and prospect of the crooked and twisted characteristics of P. yunnanensis stem. For Invent Plan..

[7] Zheng-li L, Yong-jun F, Ke-ming C (1994). Comparative anatomical observations of wood structures of Pinus yunnanensis and P. yunnanensis var. pygmaea. J Integr Plant Biol.

[8] Zlobin IE, Vankova R, Dobrev PI, Gaudinova A, Kartashov AV, Ivanov YV, Ivanova AI, Kuznetsov VV (2023). Abscisic Acid and Cytokinins Are Not Involved in the Regulation of Stomatal Conductance of Scots Pine Saplings during Post-Drought Recovery. Biomol.

[9] Shi Y, Ding Y, Yang S (2015). Cold signal transduction and its interplay with phytohormones during cold acclimation. Plant Cell Physiol.

[10] Ilyas M, Nisar M, Khan N, Hazrat A, Khan AH, Hayat K (2021). Drought tolerance strategies in plants: a mechanistic approach. J Plant Growth Regul.

[11] Chen H, Quan W, Liu H, Ding G (2022). Effects of Suillus luteus and S. bovinus on the physiological response and nutrient absorption of Pinus massoniana seedlings under phosphorus deficiency. Plant and Soil.

[12] Stocker T, et al. Climate Change 2013: The Physical Science Basis. Contribution of Working Group I to the Fifth Assessment Report of the Intergovernmental Panel on Climate Change. Cambridge and New York: Cambridge University Press; 2013. p. 1535.

[13] Mantova M, Herbette S, Cochard H, Torres-Ruiz JM (2022). Hydraulic failure and tree mortality: from correlation to causation. Trends Plant Sci.

[14] Dong S, Jiang Y, Dong Y, Wang L, Wang W, Ma Z (2019). A study on soybean responses to drought stress and rehydration. Saudi J Biol Sci.

[15] Li LL, Li Y, Ding GJ (2022). Research progress on effects of high temperature and drought on carbon metabolism in woody plants. J Mt Agric Biol.

[16] Yang X, Lu M, Wang Y, Wang Y, Liu Z, Chen S (2021). Response mechanism of plants to drought stress. Horticulturae.

[17] Nadal-Sala D, Grote R, Birami B, Knüver T, Rehschuh R, Schwarz S, Ruehr NK (2021). Leaf shedding and non-stomatal limitations of photosynthesis mitigate hydraulic conductance losses in scots pine saplings during severe drought stress. Front Plant Sci.

[18] Samuelson LJ, Stokes TA, Ramirez MR, Mendonca CC (2019). Drought tolerance of a Pinus palustris plantation. Forest Ecol Manage.

[19] Hasan MM, Gong L, Nie Z-F, Li F-P, Ahammed GJ, Fang X-W (2021). ABA-induced stomatal movements in vascular plants during dehydration and rehydration. Environ Exp Bot.

[20] Pashkovskiy PP, Vankova R, Zlobin IE, Dobrev P, Kartashov AV, Ivanova AI, Ivanov VP, Marchenko SI, Nartov DI, Ivanov YV (2022). Hormonal responses to short-term and long-term water deficit in native Scots pine and Norway spruce trees. Environ Exp Bot.

[21] Kartashov AV, Zlobin IE, Pashkovskiy PP, Pojidaeva ES, Ivanov YV, Ivanova AI, Ivanov VP, Marchenko SI, Nartov DI, Kuznetsov VV (2023). Effects of drought stress memory on the accumulation of stress-protective compounds in naturally grown pine and spruce. Plant Physiol Biochem.

[22] Pérez-Oliver MA (2023). González-Mas MdC, Renau-Morata B, Arrillaga I, Sales E: Heat-Priming during Somatic Embryogenesis Increased Resilience to Drought Stress in the Generated Maritime Pine (Pinus pinaster) Plants. Int J Mol Sci.

[23] Guo Y, Zhang S, Ai J, Zhang P, Yao H, Liu Y, Zhang X (2023). Transcriptomic and biochemical analyses of drought response mechanism in mung bean (Vignaradiata (L.) Wilczek) leaves. Plos one..

[24] Fox H, Doron-Faigenboim A, Kelly G, Bourstein R, Attia Z, Zhou J (2018). Transcriptome analysis of Pinus halepensis under drought stress and during recovery. Tree Physiol.

[25] Chutipaijit S (2016). Changes in physiological and antioxidant activity of indica rice seedlings in response to mannitol-induced osmotic stress. Chilean J Agri Res.

[26] Luo Y, Hu T, Huo Y, Wang L, Zhang L, Yan R (2023). Transcriptomic and physiological analyses reveal the molecular mechanism through which exogenous melatonin increases drought stress tolerance in chrysanthemum. Plants.

[27] Zhang A, Liu M, Gu W, Chen Z, Gu Y, Pei L, Tian R (2021). Effect of drought on photosynthesis, total antioxidant capacity, bioactive component accumulation, and the transcriptome of Atractylodes lancea. BMC Plant Biol.

[28] Byeon S, Kim S, Hong J, Kim TK, Huh W, Kim K, et al. Drought hardening effect on improving transplant stress tolerance in Pinus densiflora. Environ Exp Bot. 2023;207:105222.

[29] Zhou J, Chen S, Shi W, David-Schwartz R, Li S, Yang F, Lin Z (2021). Transcriptome profiling reveals the effects of drought tolerance in Giant Juncao. BMC Plant Biol.

[30] Li M, Wang H, Zhao X, Lu Z, Sun X, Ding G (2021). Role of Suillus placidus in improving the drought tolerance of Masson Pine (Pinus massoniana Lamb.) seedlings. Forests..

[31] Rhoads A, Au KF (2015). PacBio sequencing and its applications. Genomics Proteomics Bioinformatics.

[32] Li J, Harata-Lee Y, Denton MD, Feng Q, Rathjen JR, Qu Z, Adelson DL (2017). Long read reference genome-free reconstruction of a full-length transcriptome from Astragalus membranaceus reveals transcript variants involved in bioactive compound biosynthesis. Cell Discov.

[33] Li W, Fu Y, Lv W, Zhao S, Feng H, Shao L, Li C, Yang J (2022). Characterization of the early gene expression profile in Populus ussuriensis under cold stress using PacBio SMRT sequencing integrated with RNA-seq reads. Tree Physiol.

[34] Wei J, Pei X, Hu X, Sun S, Zhao C, Han R (2022). Applications of transcriptome in conifer species. Plant Cell Tissue Organ Cult (PCTOC).

[35] Jiang C, Li X, Zou J, Ren J, Jin C, Zhang H, Yu H, Jin H (2021). Comparative transcriptome analysis of genes involved in the drought stress response of two peanut (Arachis hypogaea L) varieties. BMC Plant Biol..

[36] de María N, Guevara MÁ, Perdiguero P, Vélez MD, Cabezas JA, López-Hinojosa M (2020). Molecular study of drought response in the Mediterranean conifer Pinus pinaster Ait: differential transcriptomic profiling reveals constitutive water deficit-independent drought tolerance mechanisms. Ecol Evol.

[37] Sánchez-Salguero R, Camarero JJ, Rozas V, Génova M, Olano JM, Arzac A (2018). Resist, recover or both? Growth plasticity in response to drought is geographically structured and linked to intraspecific variability in Pinus pinaster. J Biogeography.

[38] Yang Y, Li C (2016). Photosynthesis and growth adaptation of Pterocarya stenoptera and Pinus elliottii seedlings to submergence and drought. Photosynthetica.

[39] Eberhardt TL, Samuelson LJ (2022). Comparison of lignin and polysaccharide sugar contents for slash, longleaf, and loblolly pine growth rings formed during periods of soil moisture extremes. Wood Sci Technol.

[40] Chen J, Zhang S, Luo T, Zheng W, Yang W, Li J, Wang Y, Wang S (2021). Distribution patterns of Pinus yunnanensis and P yunnanensis var pygmaea and related key ecological factors. J Northeast For Univ..

[41] Wang J, Yu Y, Jiang C, Sun Z, Wang X, Wang Z, Ren J, Wang Z, Wang X, Yang Z (2023). Comparative analysis of physiology-anatomy and transcriptome-metabolome involving acute drought stress response of root between two distinct peanut cultivars at seedling stage. Environ Exp Bot.

[42] Talbi S, Rojas JA, Sahrawy M, Rodríguez-Serrano M, Cárdenas KE, Debouba M, Sandalio LM (2020). Effect of drought on growth, photosynthesis and total antioxidant capacity of the saharan plant Oudeneya africana. Environ Exp Bot.

[43] Hartmann H, Ziegler W, Kolle O, Trumbore S (2013). Thirst beats hunger–declining hydration during drought prevents carbon starvation in Norway spruce saplings. New Phytol.

[44] Signori-Müller C, Oliveira RS, Barros FV, Tavares JV, Gilpin M, Diniz FC, Zevallos MJM, Yupayccana CAS, Acosta M, Bacca J (2021). Non-structural carbohydrates mediate seasonal water stress across Amazon forests. Nat Commun.

[45] Guo X, Peng C, Li T, Huang J, Song H, Zhu Q, Wang M (2021). The effects of drought and re-watering on non-structural carbohydrates of Pinus tabulaeformis seedlings. Biology.

[46] Galiano L, Timofeeva G, Saurer M, Siegwolf R, Martínez-Vilalta J, Hommel R, Gessler A (2017). The fate of recently fixed carbon after drought release: towards unravelling C storage regulation in Tilia platyphyllos and Pinus sylvestris. Plant, Cell Environ.

[47] Chandrasekaran U, Byeon S, Kim K, Kim SH, Park CO, Lee Y-S, Kim HS (2022). Short-term severe drought influences root volatile biosynthesis in eastern white pine (Pinus strobus L). Front Plant Sci.

[48] Lemoine R, Camera SL, Atanassova R, Dédaldéchamp F, Allario T, Pourtau N, Bonnemain J-L, Laloi M, Coutos-Thévenot P, Maurousset L (2013). Source-to-sink transport of sugar and regulation by environmental factors. Front Plant Sci.

[49] Li XH, Xia ZL, Yu QW, Wang J, Wang ZW, Wu D (2019). Evaluation essay for drought tolerance of tobacco varieties at different developmental stages. J Mt Agric Biol.

[50] Li P, Lin P, Zhao Z, Li Z, Liu Y, Huang C, Huang G, Xu L, Deng Z, Zhang Y (2022). Gene co-expression analysis reveals transcriptome divergence between wild and cultivated sugarcane under drought stress. Int J Mol Sci.

[51] Cheng S-B, Yang X-Z, Zou L, Wu D-D, Lu J-L, Cheng Y-R, Wang Y, Zeng J, Kang H-Y, Sha L-N (2022). Comparative physiological and root transcriptome analysis of two annual ryegrass cultivars under drought stress. J Plant Physiol.

[52] Shao C, Duan H, Ding G, Luo X, Fu Y, Lou Q (2022). Physiological and biochemical dynamics of Pinus massoniana Lamb seedlings under extreme drought stress and during recovery. Forests..

[53] Su Y, Jiao M, Guan H, Zhao Y, Deji C, Chen G (2023). Comparative transcriptome analysis of Saposhnikovia divaricata to reveal drought and rehydration adaption strategies. Mol Biol Rep.

[54] Chen Y, Li C, Yi J, Yang Y, Lei C, Gong M (2019). Transcriptome response to drought, rehydration and re-dehydration in potato. Int J Mol Sci.

[55] Feng X, Yang Z, Wang X (2021). Tissue-specific transcriptome analysis of drought stress and rehydration in Trachycarpus fortunei at seedling. PeerJ.

[56] Lei P, Liu Z, Li J, Jin G, Xu L, Ji X, Zhao X, Tao L, Meng F (2022). Integration of the physiology, transcriptome and proteome reveals the molecular mechanism of drought tolerance in cupressus gigantea. Forests.

[57] Liu C, Duan N, Chen X, Li H, Zhao X, Duo P, Wang J, Li Q (2022). Metabolic pathways involved in the drought stress response of Nitraria tangutorum as revealed by transcriptome analysis. Forests.

[58] Chen X, Chen H, Xu H, Li M, Luo Q, Wang T, Yang Z, Gan S (2023). Effects of drought and rehydration on root gene expression in seedlings of Pinus massoniana Lamb. Tree Physiol.

[59] Li X, Liu L, Sun S, Li Y, Jia L, Ye S, Yu Y, Dossa K, Luan Y (2022). Leaf-transcriptome profiles of phoebe bournei provide insights into temporal drought stress responses. Front Plant Sci.

[60] Zhang Y, Diao S, Ding X, Sun J, Luan Q, Jiang J (2023). Transcriptional regulation modulates terpenoid biosynthesis of Pinus elliottii under drought stress. Ind Crops Prod.

[61] Rabeh K, Sbabou L, Rachidi F, Ferradouss A, Laghmari G, Aasfar A, Arroussi HE, Ouajdi M, Antry SE, Belkadi B (2023). Lipidomic Profiling of Argania spinosa L (Skeels) Following Drought Stress. Appl Biochem Biotechnol..

[62] Wu C, Wang Y, Sun H. Targeted and untargeted metabolomics reveals deep analysis of drought stress responses in needles and roots of Pinus taeda seedlings. Front Plant Sci. 2022;13:1031466. 10.3389/fpls.2022.1031466 PMC9927248 36798806

[63] Xiao F, Zhao Y, Wang X-R, Liu Q, Ran J (2021). Transcriptome analysis of needle and root of Pinus massoniana in response to continuous drought stress. Plants.

[64] Joshi-Saha A, Valon C, Leung J (2011). A brand new START: abscisic acid perception and transduction in the guard cell. Sci Signal..

[65] Agurla S, Gahir S, Munemasa S, Murata Y, Raghavendra AS (2018). Mechanism of stomatal closure in plants exposed to drought and cold stress. Adv Exp Med Biol.

[66] Li W, Lee J, Yu S, Wang F, Lv W, Zhang X, Li C, Yang J (2021). Characterization and analysis of the transcriptome response to drought in Larix kaempferi using PacBio full-length cDNA sequencing integrated with de novo RNA-seq reads. Planta.

[67] Meng H-L, Sun P-Y, Wang J-R, Sun X-Q, Zheng C-Z, Fan T, Chen Q-F, Li H-Y (2022). Comparative physiological, transcriptomic, and WGCNA analyses reveal the key genes and regulatory pathways associated with drought tolerance in Tartary buckwheat. Front Plant Sci.

[68] Park S-Y, Fung P, Nishimura N, Jensen DR, Fujii H, Zhao Y, Lumba S, Santiago J, Rodrigues A (2009). Chow T-fF: Abscisic acid inhibits type 2C protein phosphatases via the PYR/PYL family of START proteins. Science.

[69] Jiang L, Zhang X., Chen Z. Structural Basis of ABA Perception by PYR/PYL/RCAR Receptors. In: Zhang DP, editor. Abscisic Acid: Metabolism, Transport and Signaling. Dordrecht: Springer; 2014. p. 117–35.

[70] Mao H, Jian C, Cheng X, Chen B, Mei F, Li F, Zhang Y, Li S, Du L, Li T (2022). The wheat ABA receptor gene TaPYL1-1B contributes to drought tolerance and grain yield by increasing water-use efficiency. Plant Biotechnol J.

[71] Zi X, Zhou S, Wu B (2022). Alpha-linolenic acid mediates diverse drought responses in maize (Zea mays l) at seedling and flowering stages. Molecules..

[72] Yang H, Zhao L, Zhao S, Wang J, Shi H (2017). Biochemical and transcriptomic analyses of drought stress responses of LY1306 tobacco strain. Sci Rep.

[73] Zhang Q, Zhao Y, Zhang J, Li X, Ma F, Duan M, Zhang B, Li H (2021). The responses of the lipoxygenase gene family to salt and drought stress in foxtail millet (Setaria italica). Life.

[74] Xing Q, Zhang X, Li Y, Shao Q, Cao S, Wang F, Qi H (2019). The lipoxygenase CmLOX13 from oriental melon enhanced severe drought tolerance via regulating ABA accumulation and stomatal closure in Arabidopsis. Environ Exp Bot.

[75] Du B, Zhao W, An Y, Li Y, Zhang X, Song L, Guo C (2019). Overexpression of an alfalfa glutathione S-transferase gene improved the saline-alkali tolerance of transgenic tobacco. Biol Open..

[76] Junaid MD, Öztürk Gökçe ZN, Gökçe AF (2023). Investigation of drought induced biochemical and gene expression changes in carrot cultivars. Mol Biol Rep.

[77] Chaudhry UK, Gökçe ZNÖ, Gökçe AF (2021). Drought and salt stress effects on biochemical changes and gene expression of photosystem II and catalase genes in selected onion cultivars. Biologia.

[78] Zhou P, An Y, Wang Z, Du H, Huang B (2014). Characterization of gene expression associated with drought avoidance and tolerance traits in a perennial grass species. PLoS ONE.

[79] Aboulela M, Nakagawa T, Oshima A, Nishimura K, Tanaka Y (2018). The Arabidopsis COPII components, AtSEC23A and AtSEC23D, are essential for pollen wall development and exine patterning. J Exp Bot.

[80] Li L, Wang Y (2023). Independent and combined influence of drought stress and nitrogen deficiency on physiological and proteomic changes of barley leaves. Environ Exp Bot.

[81] Re R, Pellegrini N, Proteggente A, Pannala A, Yang M, Rice-Evans C (1999). Antioxidant activity applying an improved ABTS radical cation decolorization assay. Free Radic Biol Med.

[82] Zhang J, Huang D, Zhao X, Zhang M (2021). Evaluation of drought resistance and transcriptome analysis for the identification of drought-responsive genes in Iris germanica. Sci Rep.

[83] Buysse J, Merckx R (1993). An improved colorimetric method to quantify sugar content of plant tissue. J Exp Bot.

[84] Hansen J, Møller I (1975). Percolation of starch and soluble carbohydrates from plant tissue for quantitative determination with anthrone. Anal Biochem.

[85] Li Mh (2008). Xiao Wf, Shi P, Wang Sg, Zhong Yd, Liu Xl, Wang Xd, Cai Xh, Shi Zm: Nitrogen and carbon source–sink relationships in trees at the Himalayan treelines compared with lower elevations. Plant, Cell Environ.

[86] Jin X, Yang X, Islam E, Liu D, Mahmood Q (2008). Effects of cadmium on ultrastructure and antioxidative defense system in hyperaccumulator and non-hyperaccumulator ecotypes of Sedum alfredii Hance. J Hazard Mater.

[87] Lichtenthaler HK, Wellburn AR (1983). Determinations of total carotenoids and chlorophylls a and b of leaf extracts in different solvents. Biochem Soc Trans.

[88] Li W, Godzik A (2006). Cd-hit: a fast program for clustering and comparing large sets of protein or nucleotide sequences. Bioinformatics.

[89] Simão FA, Waterhouse RM, Ioannidis P, Kriventseva EV, Zdobnov EM (2015). BUSCO: assessing genome assembly and annotation completeness with single-copy orthologs. Bioinformatics.

[90] Wu J-J, Han Y-W, Lin C-F, Cai J, Zhao Y-P (2023). Benchmarking gene set of gymnosperms for assessing genome and annotation completeness in BUSCO. Horticulture Res..

[91] Huerta-Cepas J, Szklarczyk D, Heller D, Hernández-Plaza A, Forslund SK, Cook H, Mende DR, Letunic I, Rattei T, Jensen LJ (2019). eggNOG 5.0: a hierarchical, functionally and phylogenetically annotated orthology resource based on 5090 organisms and 2502 viruses. Nucleic Acids Res..

[92] Beier S, Thiel T, Münch T, Scholz U, Mascher M (2017). MISA-web: a web server for microsatellite prediction. Bioinformatics.

[93] Chen S, Zhou Y, Chen Y, Gu J (2018). fastp: an ultra-fast all-in-one FASTQ preprocessor. Bioinformatics.

[94] Langmead B, Salzberg SL (2012). Fast gapped-read alignment with Bowtie 2. Nat Methods.

[95] Li B, Dewey CN (2011). RSEM: accurate transcript quantification from RNA-Seq data with or without a reference genome. BMC Bioinformatics.

[96] Love MI, Huber W, Anders S (2014). Moderated estimation of fold change and dispersion for RNA-seq data with DESeq2. Genome Biol.

[97] Wu T, Hu E, Xu S, Chen M, Guo P, Dai Z, Feng T, Zhou L, Tang W, Zhan L (2021). clusterProfiler 4.0: A universal enrichment tool for interpreting omics data. Innovation (Camb)..

[98] Ernst J, Bar-Joseph Z (2006). STEM: a tool for the analysis of short time series gene expression data. BMC Bioinformatics.

[99] Langfelder P, Horvath S (2008). WGCNA: an R package for weighted correlation network analysis. BMC Bioinformatics.

[100] Livak KJ, Schmittgen TD (2001). Analysis of relative gene expression data using real-time quantitative PCR and the 2− ΔΔCT method. Methods.

[101] Lê S, Josse J, Husson F (2008). FactoMineR: an R package for multivariate analysis. J Stat Softw.

