# Supplementary material for: Physiological responses to drought stress of three pine species and comparative transcriptome analysis of Pinus yunnanensis var. pygmaea
Source: BMC Genomics. 2024 Mar 16;25:281. doi: 10.1186/s12864-024-10205-5 (PMC10944613; doi:10.1186/s12864-024-10205-5)
Supplement: Supplementary file 1 — Supplementary Material 1. [file 12864_2024_10205_MOESM1_ESM.docx]

## Supplementary material

Table S1. The primer list of qRT-PCR.

| GeneID | Primers(5'-3') |
| --- | --- |
| Actin(+) | GCGGATATGGTGTTTCGCTG |
| Actin(-) | AGAACTGCACCTCCCAGGTA |
| D1_transcript_119551(+) | GCCATCTTCACACAACTGCG |
| D1_transcript_119551(-) | CCCTCGTCAAATCCAGCCTT |
| D1_transcript_51433(+) | GGATGGGGCTCCTTATCTGC |
| D1_transcript_51433(-) | ATCCCTGCCTGGTACTCCAT |
| D1_transcript_119121(+) | AAATCTGGTACGAGCGCACA |
| D1_transcript_119121(-) | GAGGAGGAGGAGGTCCCATT |
| D1_transcript_72349(+) | GTGTGATGGTTGCTGCCAAG |
| D1_transcript_72349(-) | TTCGGAGCCTTCATCCAACC |
| D1_transcript_100815(+) | ACTCAAACTGCAGCCCATCA |
| D1_transcript_100815(-) | TGCTGACACCCCCAACTTTT |
| D1_transcript_55990(+) | ACATGGATGGCTATGGCGTT |
| D1_transcript_55990(-) | CTACCTCCCACGATCACAGC |
| D1_transcript_57940(+) | GACCCCAGTGATTGTTCGGT |
| D1_transcript_57940(-) | CGCCATGGCTCTTGAATGTG |

Table S2. Annotated transcription factors family types and quantities.

| Family | n |
| --- | --- |
| bHLH | 2650 |
| NAC | 1832 |
| MYB_related | 1642 |
| ERF | 1408 |
| C3H | 1334 |
| C2H2 | 1062 |
| WRKY | 1013 |
| bZIP | 925 |
| B3 | 863 |
| MYB | 776 |
| FAR1 | 765 |
| G2-like | 741 |
| Trihelix | 619 |
| GRAS | 519 |
| M-type_MADS | 504 |
| LBD | 472 |
| GATA | 404 |
| HSF | 396 |
| HB-other | 383 |
| ARF | 377 |
| HD-ZIP | 372 |
| GeBP | 310 |
| NF-YA | 300 |
| TCP | 295 |
| MIKC_MADS | 272 |
| Nin-like | 243 |
| NF-YC | 242 |
| NF-YB | 213 |
| BES1 | 195 |
| STAT | 195 |
| CO-like | 191 |
| Dof | 190 |
| SBP | 188 |
| CAMTA | 186 |
| E2F/DP | 177 |
| BBR-BPC | 147 |
| TALE | 146 |
| DBB | 139 |
| ZF-HD | 131 |
| AP2 | 123 |
| CPP | 110 |
| ARR-B | 102 |
| YABBY | 102 |
| S1Fa-like | 98 |
| WOX | 80 |
| GRF | 64 |
| EIL | 53 |
| SRS | 52 |
| LFY | 49 |
| HRT-like | 47 |
| Whirly | 47 |
| NF-X1 | 46 |
| HB-PHD | 41 |
| VOZ | 41 |
| LSD | 40 |
| RAV | 24 |
| SAP | 7 |
| NZZ/SPL | 2 |
